# Nanomaterial-Assisted Signal Enhancement of Hybridization for DNA Biosensors: A Review

**DOI:** 10.3390/s90907343

**Published:** 2009-09-11

**Authors:** Jinhuai Liu, Jinyun Liu, Liangbao Yang, Xing Chen, Meiyun Zhang, Fanli Meng, Tao Luo, Minqiang Li

**Affiliations:** The Key Laboratory of Biomimetic Sensing and Advanced Robot Technology, Institute of Intelligent Machines, Chinese Academy of Sciences, Hefei 230031, China

**Keywords:** DNA detection, sensitivity, nanosemiconductors, quantum-dots, nanopaticles, carbon nanotubes

## Abstract

Detection of DNA sequences has received broad attention due to its potential applications in a variety of fields. As sensitivity of DNA biosensors is determined by signal variation of hybridization events, the signal enhancement is of great significance for improving the sensitivity in DNA detection, which still remains a great challenge. Nanomaterials, which possess some unique chemical and physical properties caused by nanoscale effects, provide a new opportunity for developing novel nanomaterial-based signal-enhancers for DNA biosensors. In this review, recent progress concerning this field, including some newly-developed signal enhancement approaches using quantum-dots, carbon nanotubes and their composites reported by our group and other researchers are comprehensively summarized. Reports on signal enhancement of DNA biosensors by non-nanomaterials, such as enzymes and polymer reagents, are also reviewed for comparison. Furthermore, the prospects for developing DNA biosensors using nanomaterials as signal-enhancers in future are also indicated.

## 1. Introduction

DNA, as the main component of gene chromosomes in cells, is of great significance for both inheritance and good health [1,2]. All of the genetic information is determined precisely by the base sequences in DNA. Any disorder in these base sequences can result in the distortion of inheritance or the occurrence of diseases [3–8]. In the past decade, detection of DNA sequences has received broad attention due to its potential applications in a variety of fields, e.g., biomedical engineering, clinical diagnosis, food development and environmental protection [9–12]. Among the various approaches, detection of DNA sequences through hybridization with complementary probe DNA is of particular interest. Traditional DNA biosensors for hybridization detection convey DNA base-pair recognition events into a readable signal containing electrochemical, electric, and weight information [13–15]. Accordingly, the detection sensitivity of DNA biosensors is determined by the signal variation of hybridization events, hence the significance of signal enhancement for improving the sensitivity of DNA detection, and in recent years, many efforts have been devoted to enhancing the hybridization signal of DNA biosensors [16–18]. In spite of these achievements, a facile highly-effective strategy with long-term stability for signal amplification still remains a challenge.

In the recent past years, nanomaterials have received increasing attention due to their unique chemical and physical properties caused by their quantum confinement, surface, small size, and macro quantum tunnel effects [19–22]. Various nanostructures, such as nanoparticles, nanowires, nanotubes, and nanospheres, have been prepared [23–29]. The enhanced performance of nanomaterial-based devices provides a new opportunity for amplifying hybridization signal of DNA biosensors by incorporating DNA with nanomaterials.

Until now, there have been several reviews of the applications of nanomaterials in DNA biosensors or DNA-based fabrication of nanomaterials [30–33]. However, there is no review focused on nanomaterial-assisted signal enhancement of hybridization for DNA biosensors. Herein, recent progress in this area, including the reports by our group and other researchers, are comprehensively summarized. Some newly-developed signal enhancement approaches by using quantum-dots, carbon nanotubes and their composites are also described. Furthermore, the prospects for developing DNA biosensors using nanomaterials as signal-enhancers are discussed.

## 2. DNA Hybridization and Signal-Detecting Methods

As we know, there are four types of bases in DNA sequences, i.e., adenine (A), guanine (G), thymine (T) and cytosine (C). Adenine can pair with thymine to produce two Watson-Crick hydrogen bonds, while guanine can pair with cytosine to form three. Their possible combination types are A-T, T-A, G-C, and C-G. Accordingly, new double-stranded DNA can be fabricated if there are some complementary bases between two DNA sequences. This process is defined as DNA hybridization. During these years, many techniques, e.g., chromatography, spectrophotometry and spectrophoto-fluorimetry, in addition to electrochemical methods, have been developed for the detection of DNA hybridization due to its significant applications in life sciences, medicine, electronics, etc. [34–37]. Among them, electrochemical methods are of particular interest worldwide because of their fascinating advantages, such as relatively excellent selectivity and sensitivity, low cost, and low-interference characteristics.

As to the hybridization signals in DNA detection, taking the DNA biosensor-based methods for instance, signals are significantly determined by the transducer, which is one of the main components in a DNA biosensor. By using transducers, base pair recognition events in DNA biosensor are converted into readable analytical signals, which can be current, frequency or fluorescence variations [38–40]. In our study, as presented below, the main efforts are focused on the DNA hybridization detection through all these measurement methods.

## 3. Hybridization Signal Enhanced by Nanomaterials

### 3.1. Nanosemiconductors

As a versatile semiconductor, ZnO has been investigated for signal enhancement in DNA hybridization detection. Zhi *et al.* developed a DNA biosensor based on micropatterned amine-functionalized ZnO/SiO_2_ core/shell nanorod arrays on nanocrystalline diamond substrates [41]. Nanostructural ZnO was served as a fluorescence enhancer. The results indicated that the as-fabricated biosensor exhibited high sensitivity and specificity in hybridization detection. Two aspects were considered as the explanations: 1) ZnO nanorod arrays possessed high specific surface areas, so that more amine groups and more probe DNA could be immobilized; 2) The presence of ZnO nanorods would change the decay rates of radiative and nonradiative pathways of the 6-carboxyfluorescein DNA oligonucleotides, leading to the decrease of fluorophore lifetime and the increase of net quantum yields which was in favor of fluorescent enhancement. These findings would greatly promote the use of nanocrystalline diamond films and ZnO nanomaterials as signal enhancing platforms for highly-sensitive DNA detection. Besides, Lee and co-workers observed a signal enhancement during the immobilization and hybridization of oligonucleotides on ZnO nanorods, indicating the potential application of ZnO nanorods for signal enhancement in DNA biosensors [42].

**Figure 1.**
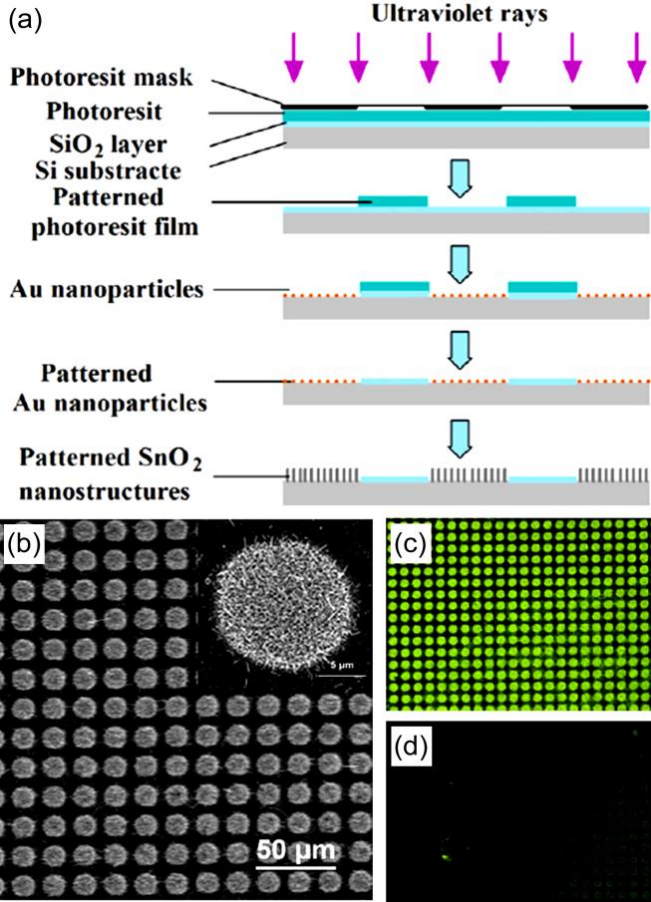
(a) Schematic illustration of the fabrication of patterned SnO_2_ nanomaterials on silicon substrate. (b) Low- and high- magnification (insert) SEM images of the patterned SnO_2_ nanomaterials. The fluorescence emission photos from (c) covalent- and (d) noncovalent-linked probe DNA. Reprinted with permission from [43]. Copyright (2008) IOP Publishing Ltd.

Recently, we have reported the finding that SnO_2_ nanomaterials could also be excellent fluorescent signal-enhancers in DNA hybridization detection [43]. In our study, SnO_2_ nanomaterials were fabricated by vapor phase transport on patterned Au nanoparticle catalysts, as shown in Figure 1. An amine group modified probe DNA at the 5′ end was covalently immobilized on the substrate. When the complementary target DNA modified with a 6-carboxyfluoresce at the 3′ end was hybridized with a probe DNA, enhanced fluorescent signal was obtained on the surface of SnO_2_ nanomaterials. The detection limit of DNA hybridization could be 1.0 × 10^−14^ M. Furthermore, by comparing the different fluorescence images from covalent and non-covalent linking schemes, the covalent method was confirmed to be more effective for immobilizing probe DNA. We also revealed that target DNA was linked on substrate by hybridization rather than physical adsorption.

Nanostructural metal oxide films were also investigated for amplifying electrochemical DNA-sensing signal. Mathur *et al.* deposited a series of nanostructural MO_x_ films, such as TiO_2_, SnO_2_ and Fe_3_O_4_, on the surface of disposable pencil graphite electrodes (PGEs) [44]. The results showed that the applications of nanostructural MO_x_ coatings on PGEs leaded to a significant enhancement of the guanine oxidation signal as compared with bare PGEs. MO_x_@PGEs as high surface area electrodes exhibited an important advantage for DNA immobilization, and could produce a high response. These findings make it possible for electrochemical detection of DNA hybridization with enhanced signal on the disposable graphite surface.

**Figure 2.**
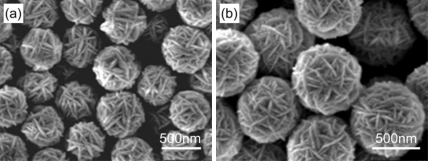
SEM images of the walnut-like CdS nanoarchitectures synthesized via a solvothermal route. Reprinted with permission from [46]. Copyright (2008) Elsevier B.V.

**Figure 3.**
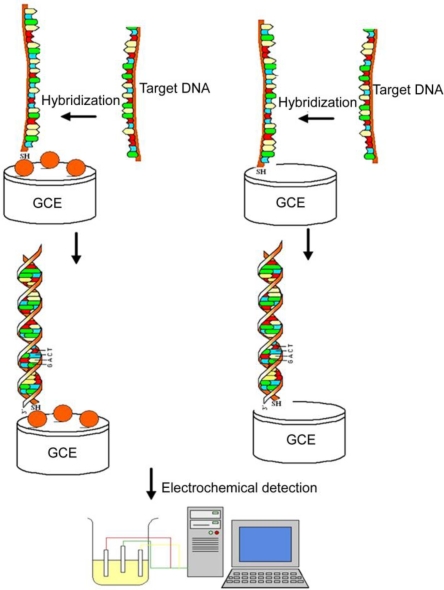
Process of CdS-modified GCE for the detection of DNA hybridization. (Left) Formation of a layer of CdS-ODN nanoconjugates on the surface of GCE before DNA hybridization. (Right) Direct mobilization of ODN on the surface of bare GCE for DNA hybridization detection. Reprinted with permission from [46]. Copyright (2008) Elsevier B.V.

On the basis of Fang’s findings, i.e., CdS nanoarchitectures coated with free carboxyl groups on the surface could covalently bind with amine-modified probe DNA to produce CdS-DNA nanoconjugates [45], our group constructed a novel DNA hybridization biosensor based on CdS modified glass carbon electrode [46]. Firstly, we synthesized a novel CdS nanoarchitecture with special walnut-like morphology (Figure 2) via a solvothermal route. Secondly, the mercapto group-linked probe DNA was covalently immobilized onto the as-prepared CdS nanomaterials and exposed to oligonucleotide (ODN) target for hybridization, as shown in Figure 3. The results showed that when target DNA hybridized with probe CdS-ODN conjugates and the double helix formed on the modified electrode, a significantly improved response could be obtained, compared with the bare electrodes. The signal was a good linear fit with the minus logarithm of target ODN concentration with a detection limit lower than 1.0 × 10^−12^ M. Finally, we considered that the combined effects of semiconducting nature, superficial character of CdS nanoarchitectures and negative charges of CdS ligand would be the explanations for the observed signal amplification.

### 3.2. Quantum-dots (QDs)

QDs are considered as a revolution in the application of fluorophores for biological labeling as compared with traditional dye-labels [47–52]. The emission of QDs is broad, and the excitation wavelength is both narrow and independent [53–58]. In other words, different fluorescence wavelengths can be simply achieved by varying the size of QDs. All colors of QDs can be excited by a single excitation source. These unique characteristics enable QDs extremely useful for multiplexed DNA detection with a high sensitivity.

In the past few years, many efforts focused on the detection of DNA biomolecules with enhanced signals by using QDs have been reported [59–61]. Wang’s group reported an alternative separation-free multiplexed detection method based on multicolor colocalization of target-specific QD nanoprobes [62]. It was indicated that this method was capable of detecting DNA hybridization at single molecule/particle level and was therefore ideal for analyzing low-abundance targets. The hybridized and unhybridized strands could be distinguished and quantified through colorimetric measurements and particle counting. Moreover, they developed an ultrasensitive biosensor based on fluorescence resonance energy transfer (FRET) which could detect DNA at low concentrations in a separation-free format [63]. The novel DNA biosensor consists of two target-specific oligonucleotide probes, which contained a reporter probe labeled with a fluorophore and a capture probe labelled with biotin, as well as a QD conjugation with streptavidins. The QD acted as both FRET energy donor and target concentrator that amplifies the target signal by confining several targets. As FRET is highly-focused and widely-used detection method for studying the interactions between biomolecules, many efforts with great advances in this field have been reported [64–70]. Sejdic *et al.* designed a simple DNA-sensing platform on the basis of the FRET between blue-luminescent CdTe QDs and dye-labeled ssDNA [71]. A cationic polymer acted as a linker to achieve efficient energy transfer from QD donor to dye acceptor. The different interaction of single-stranded and double-stranded DNA with CdTe resulted in different changes of FRET efficiency. These findings provide a homogeneous DNA assay that has all the advantages of solution-based fluorescence detection with only minimal DNA modification. Recently, Fan’s group reported a facile approach for quickly synthesizing compact QD-DNA probes for sensitive DNA detection using FRET [72]. The radius of final QDs was only around 3 nm after applying the functional coating, resulting in a highly efficient energy transfer. Nearly 70% transfer efficiency could be achieved with only a few DNA molecules on each QD and that the FRET-based DNA detection could be performed in few minutes with a detection limit lower than 1.0 × 10^−9^ M.

Recently, more and more QD-assisted DNA biosensors with highly-enhanced sensitivity have been developed. Ozkan *et al.* fabricated some multicolour hybrid DNA probes employing green, yellow and orange color quantum dot conjugated molecular beacons with black hole quencher [73]. Target DNA identification was observed to be highly-sensitive, up to 8 ng in gel electrophoresis. Furthermore, compared with the conventional organic dye, it was revealed that the novel hybrid nanoprobes exhibited more stable performance with less background signal. Gu *et al.* used DNA functionalized quantum dots and magnetic beads (MBs) to produce fluorescence signal and isolate the QD-MB complex, which was linked via a target DNA [74]. They created a new concept for the enhanced fluorescence detection of sequenced oligonucleotides by using DNA functionalized QDs and MBs. Besides, this QD-MB based fluorescence detection was specific for only selected DNA sequences and nonresponsive to non-complementary sequences.

Different from the applications of QDs from single compound, Knoll *et al.* developed a versatile approach to designing functionalized nanotubes (NTs) with a cascaded-energy-transfer architecture by incorporating graded-bandgap ZnCdSe alloy QDs into ordered porous alumina membranes [75]. The incorporation of QDs into NTs was performed by layer-by-layer deposition. In order to evaluate energy transfer efficiency from the QDs to the Cy5 (a reactive water soluble fluorescent dye of the cyanine dye family), the changes of the PL emission spectra induced by hybridization were analyzed. They found that the photoluminescence (PL) emission intensity was reduced by ca. 3.2% after DNA hybridization, indicating that the energy transfer occurred from QDs to Cy5 in an efficiency of ca. 3.2%. As the energy transfer efficiency significantly depended on the distance between donors and acceptors, it was indicated that the efficiency from QDs to Cy5 in NTs could be further increased by using shorter chain length DNA or tuning the QD emission. Besides, an enhancement factor of ca. 15 was found for the sensitivity in such types of NTs, suggesting that NTs containing a cascaded-energy-transfer architecture could be potentially applied for the detection of trace amount of DNA.

### 3.3. Nanoparticles (NPs)

NPs-assisted signal enhancement for DNA biosensors have been developed for many years [76–80]. Au and Ag NPs are of particular interests due to their advantageous properties, such as hydrophilicity, standard fabrication methods, excellent biocompatibility, unique characteristics in the conjugation with biological recognition elements and multiplex capacity for signal transducer. Therefore, it would be the best choice to use Au or Ag NPs modified DNA as probe in DNA detection.

He *et al.* used AuNPs as mass amplifiers to improve the frequency signal of quartz crystal microbalance in DNA detection [81]. They found that the frequency shift of the indirect-labelling method was much more significant than that of the direct-labelling one. In both one-time and two-times amplification, the one-base-mismatched DNA and complementary DNA could be distinguished clearly from background. By combining the AuNPs enhancement principle of photoelectrochemical cell with their unique biocompatibility, Li’s group developed a photoelectrochemical method for quantitative detection of linear DNA hybridization using AuNP-modified DNA as probes [82]. Compared with un-modified probe, the AuNP-labeled process could lead to an obvious signal change, and thereby the sensitivity in DNA hybridization detection could be effectively improved. Jiang *et al.* demonstrated the enhancement of DNA immobilization and hybridization on gold electrode modified by AuNPs [83]. The hybridization amount of target DNA could be increased several times for manifold nanogold electrodes. The detection limit of nanogold electrode for complementary oligonucleotide and long sequence oligonucleotide could reach a concentration of 10^−9^ and 10^−11^ M, respectively, which were far more sensitive than that of the planar electrode.

More recently, Yang’s group reported a novel strategy for AuNPs-based signal enhancement by the improvement of electrocatalytic activity of labels [84]. It is known that the electrocatalytic activity of labels can be enhanced by electrochemical treatment. However, when the distance between an electrocatalytic label and an electrode is too long, the enhancement by electrochemical treatment requires extremely applied potentials, which would damage biosensing layers on the electrode. To overcome this problem, they made some efforts on a simple chemical treatment of AuNPs by using NaBH_4_ instead of electrochemical treatment. The results showed that NaBH_4_ treatment could significantly enhance electrocatalytic activity of DNA-conjugated AuNPs without damage of biosensing layers.

As the sensitivity of DNA biosensors can be greatly improved by AuNPs as amplifiers, and the effect increases as the particle size increases, when the size of AuNPs increases further to a certain size, the detection limitation will reach a critical value. Aim to investigate the potential explanation, Jiang *et al.* studied the signal enhancement by AuNPs surface modification [85]. They modified the surface of quartz crystal microbalance sensor by 12 nm NPs. It was found that the DNA immobilization amount on the nanoparticle surface was much higher than that on the plane surface, and AuNPs in a size larger than 50 nm could continuously act as amplifiers to enhance the DNA detection sensitivity.

**Figure 4.**
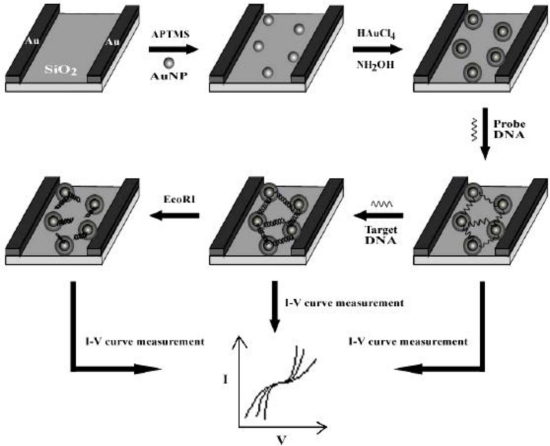
Schematic illustration of *in situ* seed-assisted growth of nanogapped AuNPs for the electrical detection of DNA hybridization. Reprinted with permission from [86]. Copyright (2008) Elsevier B.V.

In recent years, our group has made some efforts on the fabrication of nanogapped AuNPs for directly electrical detection of DNA [86]. Through a facile one-step reaction between HAuCl_4_ and NH_2_OH, the distance between neighboring AuNPs could be less than the length of probe DNA, so that the DNA strand could bridge the AuNPs to provide an electron tunneling path between microelectrodes, as shown in Figure 4.

**Figure 5.**
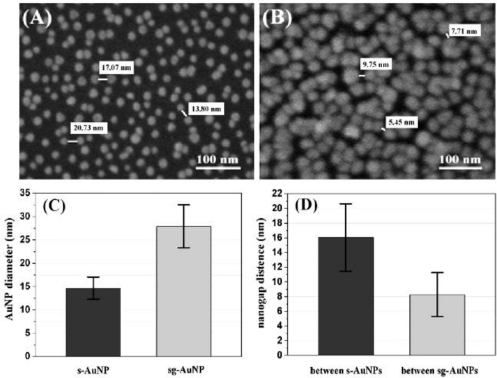
SEM images of AuNPs and the calculated distance between them: (A) single-layered AuNPs (s-AuNPs) between electrodes before growth; (B) high-magnification view of in situ seed-assisted growing (sg-AuNPs) between electrodes after 2 min growth; (C) the nanoparticle diameters of s-AuNPs and sg-AuNPs; (D) the distance of nanogap between each AuNP. Reprinted with permission from [86]. Copyright (2008) Elsevier B.V.

**Figure 6.**
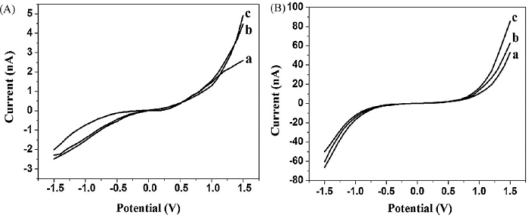
Electrical detection of DNA: (A) I–V curves of s-AuNPs: (a) bare s-AuNPs, (b) s-AuNPs with single-stranded DNA (ss-DNA, probe DNA), and (c) s-AuNPs with double-stranded DNA (ds-DNA); (B) I–V curves of sg-AuNPs: (a) bare sg-AuNPs, (b) sg-AuNPs with ss-DNA, and (c) sg-AuNPs with ds-DNA. Reprinted with permission from [86]. Copyright (2008) Elsevier B.V.

In our study, *in situ* seed-assisted growing AuNPs (sg-AuNPs) microelectrode (Figure 5) could effectively identify double-stranded DNA (dsDNA) which was immobilized on the surface of sg-AuNPs via hybridization between probe and target DNA, as shown in Figure 6.

**Figure 7.**
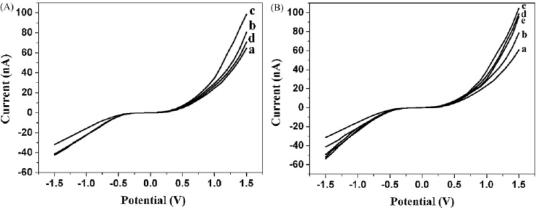
Electrical detection of DNA-EcoRI interaction: (A) I-V measurements for dsDNA cleavage by EcoRI: (a) bare sg-AuNPs, (b) sg-AuNPs with ss-DNA, (c) sg-AuNPs with ds-DNA, (d) dsDNA with EcoRI. (B) I–V measurements for assaying the cleavage specificity of EcoRI endonuclease: (a) bare sg-AuNPs, (b) sg-AuNPs with ss-DNA (probe DNA3), (c) sg-AuNPs with ds-DNA, (d) dsDNA with EcoRI, and (e) dsDNA with enzyme reaction buffer. Reprinted with permission from [86]. Copyright (2008) Elsevier B.V.

Seen from Figure 6, the current of sg-AuNPs is significantly higher than that of single-layered AuNPs (s-AuNPs), which could be ascribed to the in situ seeding growth of s-AuNPs providing more chances for electrons to tunnel between microelectrodes. Furthermore, as presented in Figure 7, I–V characteristic was also applied to distinguish dsDNA-EcoRI interaction. If dsDNA was cleaved by restriction endonuclease EcoRI, the electron tunneling path made by DNA strand was also cut off. Our findings provide a rapid and effective method for fabricating nanogapped AuNPs for label-free and direct detection of DNA and EcoRI endonuclease. Besides, it builds up a new platform for investigating biological processes.

**Figure 8.**
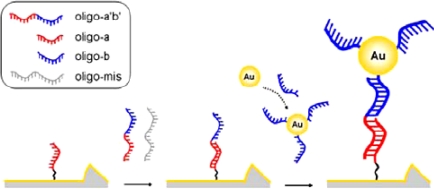
Sketch diagram of the microcantilever DNA biosensor using AuNPs as signal-enhancers.

More recently, based on the as-developed nanogapped AuNPs platform, we have fabricated a microcantilever DNA biosensor by using AuNPs as signal enhancer, as shown in Figure 8. In DNA detection, HS (CH_2_)6-3′-ATGCCTCAACTCT-5′, HS (CH_2_)6-5′-CGCATTCAGGAT-3′, and 5′-TACGAGTTGAGAATCCTGAATGCG-3′ were employed as probe DNA 1, probe DNA 2, and target DNA, respectively. When Au thin film-coated microcantilever was immobilized with probe DNA1 and MCH, the syntonic frequency would shift ca. 502 Hz. And then, after adding AuNPs-modified probe DNA2 directly, the syntonic frequency shift would be ca. 232 Hz. It was ascribed to the adsorption of AuNPs-modified probe DNA2 on the surface of microcantilever, which could be reduced effectively by using MCH before hybridization. Through a sandwich-like hybridization among probe DNA1, AuNPs-modified DNA2 and target DNA, a syntonic frequency shift of ca. 2,278 Hz was obtained. In the sandwich-like hybridization process, firstly, target DNA was combined with microcantilever by the partial hybridization between probe DNA1 and target DNA; secondly, the remained part in target DNA hybridized with AuNPs-modified probe DNA2, leading to the combination of AuNPs as signal-enhancers. In our study, the concentration of target DNA could be 1.0 × 10^−16^ M, indicating the highly-sensitive performance of such microcantilever DNA biosensor.

AgNPs have also attracted broad attention for signal enhancement of DNA biosensors in recent years, because of their quantum characteristics of small granule diameter, large specific surface area, as well as their ability to quickly transfer photoinduced electrons at the surfaces of colloidal particles [87]. In addition, AgNPs exhibit intense adsorption in the visible region, due to the surface plasmon resonance band caused by the collective excitation of conduction band electrodes [88]. *N*-(2-mercapto-propionyl) glycine-coated AgNPs have been prepared and displaced by thiolate single-stranded oligonucleotides through a ligand exchange reaction by Lakowicz and co-workers [89]. The oligonucleotide-displaced particles were further coupled with fluorophore-labeled complementary oligonucleotides by hybridization. Both the oligonucleotide-displaced and hybridized particles aggregated through electrostatic interaction with KCl in water. It is found that when the complementary oligonucleotides were fluorescein-labeled, the luminescence would be enhanced at low KCl concentrations and reduced at high KCl concentrations. Then, they revealed that the fluorophores on AgNPs showed a surface-enhanced fluorescence depending on the size of metallic cores. The coupled particles were aggregated due to multiple displacements of oligonucleotides on each particle, resulting in a strong fluorescence [90]. Furthermore, they used surface-binded AgNPs to improve the sensitivity of DNA microarrays [91]. Ag films grown on glass microscope slides were used as substrates for metal-enhanced fluorescence DNA arrays. They found that fluorescence enhancement was determined by the DNA spotting concentration. Recently, Yu’s group demonstrated a signal-amplified electrochemical immunoassay on the basis of the biocatalytic deposition of AgNPs [92]. By combining the highly-catalytic effect of enzyme and nanoparticles, the sensitivity of immunoassay was enhanced dramatically, indicating that this approach would be a promising strategy to achieve a lower detection limit for DNA biosensors.

Besides the traditional strategies for signal enhancement of DNA biosensors by Au and Ag NPs, silica nanoparticles have also been investigated as potential signal-enhancers in recent years. Xia *et al.* constructed a silica nanoparticle-based interface for sensitive sequence-specific detection of DNA [93]. They demonstrated that the silanized silica nanoparticles could be easily assembled on a self-assembled monolayer of cysteine on a gold electrode to form a monolayer interface which prominently enhanced the DNA adsorption. It could be ascribed to the large surface area of particles and the surface-chemistry-induced weak interactions between nanoparticles and biomolecules. These findings provide a novel route for fabricating electrochemical DNA biosensors which are sensitive for sequence-specific DNA detection.

### 3.4. Carbon nanotubes (CNTs) and their composites

Since the discovery of CNTs by Iijima in 1991 [94], they have received much attention due to their unique electronic, chemical, and mechanical properties. CNTs can promote electron-transfer reactions and improve sensitivity in electrochemistry, and thereby they are widely used in electronic devices. Modification of DNA biosensors using CNTs for signal-enhancement has become another focused field. Various CNT-assisted strategies for improving the sensitivity of DNA biosensors have been reported [95–97]. Zhang’s group described a sensitive electrochemical DNA biosensor based on multi-walled CNTs (MWCNTs) functionalized with a carboxylic acid group for covalent DNA immobilization and hybridization detection [98]. Compared with DNA sensors with oligonucleotides directly incorporated on glassy carbon electrodes, the CNT-based assay with large surface area and good charge-transport characteristics could greatly increase the quantity of attached DNA and complementary ssDNA, and thereby could improve the detection sensitivity.

Compared with the pure CNTs, CNT composites exhibit some enhanced performance as applied in DNA biosensors. Jiao *et al.* reported an electrochemical DNA biosensor based on MWCNTs/nanostructural ZnO/chitosan composites for DNA immobilization and hybridization detection [99]. The sensitivity of this DNA biosensor was explored by using the immobilized probe to hybridize with PAT gene target sequence at different concentrations. It was found that the as-fabricated biosensor could effectively discriminate different DNA sequences related to PAT gene in transgenic corn, with a detection limit of 2.8 × 10^−12^ M. As compared with the previous CNTs-based DNA biosensors, this composite matrix combined the attractive biocompatibility of ZnO nanoparticles and excellent electron-transfer ability of MWCNTs with the membrane-forming ability of chitosan, which could enhance the DNA attachment in detection.

**Figure 9.**
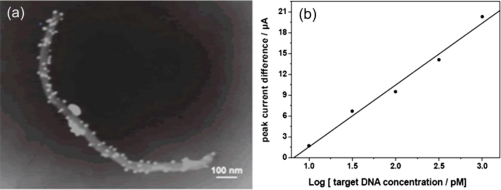
(a) TEM image of the Au/MWCNT nanocomposites. (b) Calibration current of the electroactive indicator (methylene blue) before and after hybridization. Differential pulse voltammetry measurements were performed at pulse amplitude of 50 mV and pulse width of 50 ms. Reprinted with permission from [100]. Copyright (2007) Taylor & Francis Group, LLC.

Recently, our group reported the application of Au/MWCNT composites for enhancing the electrochemical detection of DNA hybridization [100]. In our study, AuNPs were deposited on the surface of MWCNTs by one-step reaction, as shown in Figure 9a. Thiolated probe DNA was immobilized onto the Au/MWCNTs-modified glass carbon electrode (GCE) through the strong gold-sulfur linkage, which could control the molecular orientation of probe DNA. On the basis of DNA detection (Figure 9b), we found that the as-prepared Au/MWCNT composites could highly improve the sensitivity of DNA biosensor due to the enhanced conductivity and increased effective surface area of the composites. Besides, it was revealed that selectivity and reproducibility of the DNA sensor were also excellent. We believe that the novel electrochemical biosensor based on Au/MWCNT composites is significant for the hybridization detection of DNA.

## 4. Signal Enhancement of DNA Biosensor Based on Non-Nanomaterials for Comparison

In the past decade, accompanying the development of nanomaterial-assisted signal enhancement approaches, some strategies without nanomaterials have also been investigated. Pappaert *et al.* reported a micro-channel flow system to investigate the potential gain in hybridization rate stemming from the induction of a convective flow past the surface of DNA microarray [101]. The analysis time could be greatly shortened without any non-specific hybridization events. This route sheds a light for developing convection-driven hybridization systems with short analysis time and low detection limit. As DNA microarrays can produce the greatest sensitivities by using concentrated samples, Bynum’s group designed a versatile microfluidics platform with two-axis centrifuge, in which fluidic chambers rotated in a planetary relationship to a radial gravitational field [102]. The thin chambers obviate the need for sample dilution, and the sensitivities and dynamic ranges can be great improved by using this instrument. In addition, Brousseau demonstrated a novel platform for label-free detection of nucleic acid by coupling the molecular reaction state to the conductivity of a transistor [103]. By using the as-fabricated platform, low concentrations of target DNA can be detected without optical labels and associated experimental restrictions completely.

As to the biochemical approaches, Wu and co-workers reported the enhancement of hybridization efficiency of DNA targets using oligonucleotide pre-hybridization [104]. They found that pre-hybridization of a DNA oligo enhanced the subsequent hybridization efficiency of the target-DNA. The efficiency could be improved when the hybridization position occurred at a site close to the substrate surface. Komatsu *et al.* developed a novel amino-modifying reagent, of which an amino group was connected with an aromatic residue by aliphatic linker [105]. The insertion of aromatic residue could increase the reactivity of amino group on oligonucleotides, and thereby improved the sensitivity of oligonucleotide array. Recently, Xu *et al.* reported that PicoGreen could be used in combination with cationic conjugated polymers to develop label-free DNA bioasensors with enhanced detection efficiency and selectivity [106]. The detection sensitivity could be enhanced greatly through resonance energy transfer using poly diiodide salt as a light harvesting complex to take advantage of its collective optical response and optical amplification effects.

In 1996, Heller’s group demonstrated the detection of hybridization by labeling the target oligonucleotide with peroxidase for the first time [107]. Since then, enzyme-based signal amplification for DNA biosensors has been a hot field. Narvaez *et al.* reported the generation of reporter sequences by labeling with an enzyme to achieve signal amplification by successive hybridization steps [108]. The sensing layer was fabricated by the covalent binding of DNA probe over redox polymer through the 3′-phosphate group, enabling the capture of target sequence. The sensitivity of DNA assay was found to be doubled after a second hybridization step. More recently, Ehlers and co-workers described a method for the detection of DNA target molecules using capture probes and subsequent signal enhancement via a uniform polymerase chain reaction [109]. The real-time detection of immuno-polymerase chain reaction was found to be sensitive and sequence-specific in DNA detection. In addition, Chen *et al.* reported a simple and rapid capillary electrophoresis-based multiple oligonucleotide detection by using three biotin-modified DNA probes [110]. As compared with the traditional methods, the hybridizations were performed in a vial with a low nonspecific affinity which made the assay quite simple. Furthermore, the sensitivity was improved accompanying with the enhancement of horseradish peroxidase.

In recent years, signal enhancement by using non-nanomaterials, such as enzymes and polymer reagents, has been a fascinated field. Traditionally, DNA biosensors based on non-nanomaterials as signal-enhancers exhibit a rapid response and a highly-selective performance. However, the nanomaterial-assisted strategies for signal enhancement show a high sensitivity, simple procedure, as well as excellent repeatability, indicating their promising applications in practice. The reports on signal-enhancement based on nanomaterials and non-nanomaterials presented above are further summarized for comparison, as shown in Table 1.

## 5. Conclusions and Perspectives

Nanomaterials, such as nanosemiconductors, QDs, NPs, CNTs and their composites, have been widely used for improving the hybridization signal of DNA biosensors. Recent advances concerning nanomaterial-assisted signal enhancement were reviewed comprehensively. ZnO, SnO_2_, and CdS with various morphologies and structures were found to be excellent fluorescent signal-enhancers in DNA hybridization detection. As to walnut-like CdS nanoarchitectures, the combined effects of semiconducting nature, surface characteristic of CdS nanoarchitectures and negative charges of CdS ligand could be the explanations for signal amplification. As compared with traditional fluorescent dyes, the emission of QDs was broad and the excitation wavelength was both narrow and independent, indicating their promising applications for multiplexed DNA detection with a high sensitivity. Based on numerous efforts devoted to develop Au and AgNPs-based signal enhancement, Au and AgNPs have been considered as one class of the most significant signal-amplifiers for DNA detection. Furthermore, CNTs and their composites were also demonstrated as effective signal-enhancers for DNA detection at low concentration.

On the basis of current progresses in the field of nanomaterial-assisted signal enhancement for DNA biosensors, it is prospected that the following aspects would be promising directions for developing in future: 1) nanosemiconductors with novel structures which are in favor of incorporation with DNA molecules; 2) QDs-based DNA biosensors with highly-selective characteristics; 3) novel nanoscale signal-amplifiers based on the combination between NPs (such as Au and AgNPs) and CNTs. Moreover, it is noteworthy that the newly-discovered and widely-focused graphene would be a potential candidate for developing novel signal-amplifiers of DNA biosensors due to its unique molecular structure.

## Figures and Tables

**Table 1.** Summary of signal-enhancement based on nanomaterials and non-nanomaterials.

**Signal enhancers**	**Signal/method**	**Sensitivity enhancement**	**Detection limit (M)**	**Reference**
SnO_2_ nanorods	fluorescence	5-fold	1.0 × 10^−12^	43
nanoscale TiO_2_, SnO_2_, and Fe_3_O_4_ films	current		2.1, 5.4, and 4.6 × 10^−8^, respectively	44
walnut-like CdS NPs	current/cyclic and differential pulse voltammetry	ca. 3-fold	6.0 × 10^−14^	46
streptavidin-functionalized QDs	fluorescent/colorimetric measurement		< 1.0 × 10^−9^	62
single-QD	fluorescence/FRET	100-fold	4.8 × 10^−15^	63
CdSe/ZnS core-shell QDs	fluorescence/FRET		< 1.0 × 10^−9^	72
CdSe-ZnS QDs and magnetic beads	fluorescence/FRET	>100-fold	5.0 × 10^−13^	74
ZnCdSe alloy QDs	fluorescence/FRET	ca. 15-fold		75
AuNPs	frequency		1.0 × 10^−14^	81
AuNPs	photocurrent	ca. 2-fold		82
AuNPs	current/cyclic voltammetry		1.0 × 10^−11^	83
AgNPs	fluorescence	>10-fold		90
AgNPs	fluorescence	28-fold		91
AgNPs	current/anodic stripping voltammetry		0.03 ng/mL (IgG)	92
MWCNTs	current/cyclic voltammetry		3.81 × 10^−11^	98
MWCNTs/ZnO/chitosan composites	current/differential pulse voltammetry		2.8 × 10^−12^	99
Au/MWCNTs	current/differential pulse voltammetry		< 1.0 × 10^−12^	100
single electron transistor (non-nanomaterials)	current		2.5 × 10^−11^	103
cationic conjugated polymers and PicoGreen (non-nanomaterials)	fluorescence		1.0 × 10^−10^	106
enzyme	current	2-fold	1.0 × 10^−9^	108
enzyme	current		1.2 × 10^−11^	110
